# The Female Vaginal Microbiome in Health and Bacterial Vaginosis

**DOI:** 10.3389/fcimb.2021.631972

**Published:** 2021-04-07

**Authors:** Xiaodi Chen, Yune Lu, Tao Chen, Rongguo Li

**Affiliations:** Department of Clinical Laboratory, Jinan Maternity and Child Care Hospital Affiliated to Shandong First Medical University, Jinan, China

**Keywords:** vaginal microbiome, bacterial vaginosis, *Gardnerella vaginalis*, *Lactobacillus*, female health

## Abstract

The vaginal microbiome is an intricate and dynamic microecosystem that constantly undergoes fluctuations during the female menstrual cycle and the woman’s entire life. A healthy vaginal microbiome is dominated by *Lactobacillus* which produce various antimicrobial compounds. Bacterial vaginosis (BV) is characterized by the loss or sharp decline in the total number of *Lactobacillus* and a corresponding marked increase in the concentration of anaerobic microbes. BV is a highly prevalent disorder of the vaginal microbiota among women of reproductive age globally. BV is confirmed to be associated with adverse gynecologic and obstetric outcomes, such as sexually transmitted infections, pelvic inflammatory disease, and preterm birth. *Gardnerella vaginalis* is the most common microorganism identified from BV. It is the predominant microbe in polymicrobial biofilms that could shelter *G. vaginalis* and other BV-associated microbes from adverse host environments. Many efforts have been made to increase our understanding of the vaginal microbiome in health and BV. Thus, improved novel and accurate diagnosis and therapeutic strategies for BV have been developed. This review covers the features of vaginal microbiome, BV, BV-associated diseases, and various strategies of diagnosis and treatment of BV, with an emphasis on recent research progresses.

## Introduction

The human body is a holobiont consisting of the host and multispecies microbes, and the interdependency among them has been progressively enhanced in the approximately half a billion years of human–microbial coevolution (Maynard et al., 2012; Lynch and Pedersen, 2016). However, the previous knowledge of microbiota in holobionts is shaped by the researches where culture-dependent methods are used to cultivate species. With the advent of new technologies, scientists reveal that biodiversity is far beyond the microbial cells cultivated with culture-dependent methods. In particular, high-throughput sequencing approach provides further understanding of the spectrum of microbial community structure. Scientists have utilized omics approaches, including metabolomics, glycomics, metaproteomics, metatranscriptomics, and metagenomics, to verify that within a habitat, microorganisms exist in dynamic, interactive, and intricate microbial communities. The oral cavity and intestinal tract have been the long-term focus of a large number of researches on the microbial communities of human bodies. Although vaginal health is influentially significant to human reproduction and public health, it has attracted less attention. In recent years, increasing emphasis has been put on the female health, specifically in relation to vaginal microbiome (Ravel et al., 2011; Fettweis et al., 2014). The vagina harbors a huge microecosystem containing billions of microbes. A systematic detection of the female reproductive tract microbial biomass was conducted by 16S rRNA gene sequencing. Data from 110 persons of reproductive age revealed that the vagina contains 10^10^–10^11^ bacteria (Chen et al., 2017).

In the ecosystem, a homeostatic and mutualistic relationship exists between the microbiota and its human host. The host provides a humid, nutritious, and warm habitat for the microbes, whereas the resident microbiota produces antimicrobial and anti-inflammatory factors. Thus, the first line of defense against nonindigenous microorganisms is established. Nevertheless, this balance can be broken by internal and/or external factors. For internal factors, such as hormonal status (Pekmezovic et al., 2019), age (Uchihashi et al., 2015), and immune system (Ma et al., 2012), the alteration of the host environment impairs its ability to control opportunistic pathogens contained in the resident microbes that could invade the human body and cause illness. External interferences, such as antibiotics (Dethlefsen et al., 2008), infections (Lloyd-Price et al., 2019), and environmental microbial exposure (Shao et al., 2019), influence the microbiota within the habitat and are potential risk factors for diseases. The variations of these internal and/or external factors lead to the breakdown of a balanced ecosystem, also known as dysbiosis. According to DNA sequencing, reduced diversity in the intestinal ecosystem is associated with disease (Manichanh et al., 2006), whereas high diversity in the vaginal ecosystem is linked to illnesses, such as bacterial vaginosis (BV) (Fredricks et al., 2005). Furthermore, BV would trigger numerous health disorders, including adverse pregnancy outcome, human immunodeficiency virus (HIV), human papillomavirus (HPV), and pelvic inflammatory disease (PID).

This review aims to describe the vaginal microbiome with regards to female health, discuss BV characteristics, present a strong association between BV and diseases, and outline the requirement for comprehensive, accurate, and advanced diagnosis and therapies to lower adverse health outcomes.

## Healthy Vaginal Microbiome

The vaginal microbiome is an intricate and dynamic microecosystem that constantly undergoes fluctuations during the female menstrual cycle and the woman’s entire life. The vaginal mucosa is made up of a stratified squamous nonkeratinized epithelium covered by cervicovaginal secretion (Pekmezovic et al., 2019). The vaginal mucosa acquires oxygen, glucose, and other nutrients from underlying submucosal tissues through diffusion due to the limited blood supply (Linhares et al., 2011). This establishes a relatively anaerobic habitat condition. The vagina houses a complex microbial community that subsists in a symbiotic relationship with the host. Thus, the indigenous environment, microorganisms, and their genomes jointly compose the entire habitat, also known as the vaginal microbiome (Marchesi and Ravel, 2015).

In women of reproductive age, physiological changes, such as changes in hormone levels, cause fluctuations in the vaginal microbiome (Hickey et al., 2012). Marked differences have been reported between non-pregnant and pregnant women in terms of the vaginal microbiome. According to the comparison results, a sharp decline in the diversity and abundance of the vaginal microbiome is observed in pregnant women. Moreover, the predominance of *Lactobacillus* spp., *Actinomycetales*, *Clostridiales*, and *Bacteroidales* is observed in pregnant women. In non-pregnant women, the predominance of *Lactobacillus* spp., *Actinobacteria*, *Prevotella*, *Veillonellaceae*, *Streptococcu*s, *Proteobacteria*, *Bifidobacteriaceae*, *Bacteroides*, and *Burkholderiales* is observed (Aagaard et al., 2012). Thus, the vaginal microbiome would change temporally in a single person. In addition, the vaginal microbiome differs largely among individuals, and the differences are due to variations in sexual activity (Noyes et al., 2018), douching (Schwebke et al., 1999), chronic stress (Culhane et al., 2002), regional disparity (Gupta et al., 2017), race (Ravel et al., 2011), and other factors (Nelson et al., 2018). Based on high-throughput sequencing studies, five community state types (CSTs) exist in terms of the vaginal microbiome. Specifically, the research on 396 North American asymptomatic women from four ethnic groups illustrates that the majority of vaginal microbiomes are dominated by single or multiple *Lactobacillus* species and are classified into five CSTs. CSTs I, II, III, and V are dominated by *L. crispatus*, *L. gasseri*, *L. iners*, and *L. jensenii*, respectively, whereas the CST IV refers to high diversity of the microbial community characterized by obligate anaerobic bacteria. High Nugent scores are usually linked to CST IV but are also observed in other CSTs. Among the five groups, the CSTs I, II, III, and V exist in 89.7% white women and 80.2% Asian women, whereas these percentages are 61.9% and 59.6% in black and Hispanic women, respectively. A shift in ethnic groups is apparent when CST IV dominated (Ravel et al., 2011). The differences in vaginal microbiome by race of women might be driven by host genetic factors, such as immune system, ligands on the surface of epithelial cell, and the quantity and components of vaginal discharge. Compared to behavioral and cultural differences, host factors might play a more crucial role in shaping the vaginal microbiome among races (Ravel et al., 2011; Gupta et al., 2017). Currently, there are few genotyping studies associated with healthy vaginal microbiome. This is an area of research that would benefit from further investigation.

*Lactobacillus* species flourish in the vaginal anaerobic environment and produce various antimicrobial compounds, such as lactic acid, hydrogen peroxide (H_2_O_2_), and bacteriocins, thereby contributing to a healthy vaginal microbiome and establishing a defense against the invading pathogens. *Lactobacillus* species are the main source of l-lactic acid and d-lactic acid that keep the pH value of the habitat lower than 4.5 (Witkin et al., 2013; Witkin and Linhares, 2017), whereas epithelial cells contribute about 20% l-lactic acid (Boskey et al., 2001). Nonetheless, the role of H_2_O_2_ in the vaginal microbiome remains controversial. Some studies have demonstrated that it has positive effects on the inhibition of the overgrowth of pathogenic microbes (Pararas et al., 2006; Atassi and Servin, 2010; Sgibnev et al., 2015). O’Hanlon et al. found that H_2_O_2_ at physiological levels displays the undetectable ability of eliminating pathogenic microbes, whereas at high levels, it shows greater antimicrobial ability toward *Lactobacillus* spp. than pathogenic microbes (O’Hanlon et al., 2011). This finding indicates that H_2_O_2_ is not a vital antimicrobial agent for maintaining the homeostasis of the vaginal microbiome. *Lactobacillus* also synthesizes bacteriocins, a type of antimicrobial peptides that can permeabilize the microbial cell membrane of nonindigenous microorganisms (Stoyancheva et al., 2014). Furthermore, they can adhere to vaginal epithelial cells and compete with other microbial cells for binding sites (Neeser et al., 2000; do Carmo et al., 2016). This finding is important, because the adhesion of pathogen to epithelial cells is the first step and a crucial prerequisite of infection (Zárate and Nader-Macias, 2006; Ribet and Cossart, 2015). Notably, the dominant *Lactobacillus* species determines the extent of vaginal ecosystem protection. For instance, dysbiosis and low stability are usually related to the vaginal microbiota dominated by *L. iners*. On the contrary, health and high stability of the vaginal community are enhanced by *L. crispatus* that provides d- and l-lactic acids (Petrova et al., 2015). Different from other *Lactobacillus* species, *L. iners* cannot generate d-lactic acid, which plays a more important role than l-lactic acid (Witkin et al., 2013; Amabebe and Anumba, 2018; Edwards et al., 2019).

## Bacterial Vaginosis

BV is a highly prevalent lower genital tract disorder among women of reproductive age globally (Javed et al., 2019). It afflicts 23%–29% of women worldwide, and $4.8 billion is spent on symptomatic BV treatment annually (Peebles et al., 2019). BV is characterized by the loss or sharp decline in the total number of *Lactobacillus* and a corresponding 100–1000 fold increase in the concentration of facultative or obligate anaerobic microbes, such as *Gardnerella, Prevotella, Atopobium, Mobiluncus, Bifidobacterium*, *Sneathia, Leptotrichia*, and some novel bacteria in *Clostridiales* order referred to as BV-associated bacteria (BVAB) 1–3 (Eschenbach, 1993; Fredricks et al., 2005; Ling et al., 2010; Srinivasan et al., 2012; Muzny et al., 2018).

### *Gardnerella* Species

*Gardnerella* is the most common microorganism identified from the vaginal samples of women with BV (Fredricks et al., 2007; Zozaya-Hinchliffe et al., 2010). It was first isolated by Leopold from the cervix swabs of women and the urine of men in 1953 (Leopold, 1953). Later, it was found to be related to BV and named *Haemophilus vaginalis* by Gardner and Dukes in 1955 (Gardner and Dukes, 1955). It is subsequently classified into the genus *Corynebacterium* (Zinnemann and Turner, 1963). Afterward, due to the results of two taxonomic researches, it was placed into a new genus *Gardnerella* and renamed *G. vaginalis* (Greenwood and Pickett, 1980; Piot et al., 1980). Until 2019, *G. vaginalis* was the only identified species in the genus *Gardnerella*. Recently, a whole genome sequence analysis was performed on 81 *Gardnerella* strains, and results revealed the existence of 13 disparate species of genus *Gardnerella*. In the following additional physiological and chemotaxonomic analyses supported by MALDI-TOF and biochemical activity studies, three new *Gardnerella* species, namely, *G. piotii*, *G. swidsinskii*, and *G. leopoldii*, were described, and the description of *G. vaginalis* was amended (Vaneechoutte et al., 2019).

The close relationship between BV and *G. vaginalis* seems to indicate that this microorganism is the sole pathogen in BV (Schwebke et al., 2014). However, *G. vaginalis* has displayed high sensitivity and low specificity when used to determine whether BV is positive or negative (Krohn et al., 1989). Healthy or asymptomatic women may also carry *G. vaginalis* (Krohn et al., 1989; Briselden and Hillier, 1990), suggesting that the presence of *G. vaginalis* in the vagina does not always result in BV. Thus, it is important to have a proper understanding of the role of *G. vaginalis* as a spectator, participant, or causative agent of BV. In numerous studies that aimed to determine the characteristics linked to virulence, various approaches are utilized to analyze the phenotypic, genotypic, and ecotypic diversity of *G. vaginalis* (**Table 1**). Based on biochemical tests, 8 and 17 biotypes of *G. vaginalis* were identified, respectively (Piot et al., 1984; Benito et al., 1986). Much effort has been devoted to the exploration of the association between its biotypes and BV, but the results remain controversial (Piot et al., 1984; Benito et al., 1986; Briselden and Hillier, 1990; Aroutcheva et al., 2001; Udayalaxmi et al., 2011). For genotyping, four clusters (A1 to A4) of *G. vaginalis* are defined in the vaginal microbiota based on 60 kDa chaperonin protein (*cpn*60) universal target region sequencing (Hill et al., 2005). Recently, *G. vaginalis* has been classified into four subgroups (A–D) with the use of cpn60 universal target sequencing (Paramel Jayaprakash et al., 2012). Using whole-genome sequencing, *G. vaginalis* can reportedly be divided into clades 1, 2, 3, and 4 (Ahmed et al., 2012), corresponding to *cpn*60-based subgroups C, B, D, and A, respectively (Schellenberg et al., 2016). The relationship between *G. vaginalis* genotypes and BV is controversial (Paramel Jayaprakash et al., 2012; Pleckaityte et al., 2012; Balashov et al., 2014; Hilbert et al., 2017; Janulaitiene et al., 2017; Shipitsyna et al., 2019). In the only study about the ecotypes of *G. vaginalis* in 2017, three ecotypes were identified as a result of the distinct gene gain/loss of specific functions based on the combination of phylogenetic structure and functional gene analysis (Cornejo et al., 2017). Overall, these efforts have contributed to the identification of the linkage between *G. vaginalis* and different states (health, asymptomatic BV, and symptomatic BV), thereby ultimately improving the approaches for the accurate diagnosis of BV.

**Table 1:** Types of *G. vaginalis*.

Type	Numbers of type	Methods	Refs
Biotype	8	Tests of β-galactosidase, lipase, and hippurate hydrolysis	(Piot et al., 1984)
	4 groups (17 biotypes)	Tests of hippurate hydrolysis, β-galactosidase and lipase, and fermentation of arabinose, galactose and xylose	(Benito et al., 1986)
Genotype	4 (A1–A4)	*cpn*60 sequencing	(Hill et al., 2005)
	4 (A–D)	*cpn*60 sequencing	(Paramel Jayaprakash et al., 2012)
	4 (1–4)	Whole-genome sequencing	(Ahmed et al., 2012)
Ecotype	3	Phylogenetic and functional analyses	(Cornejo et al., 2017)

*G. vaginalis* harbors a variety of virulence factors associated with pathogenic potential, wherein sialidase and vaginolysin are the most widely investigated factors (Gelber et al., 2008; Santiago et al., 2011; Zilnyte et al., 2015; Hardy et al., 2017b; Robinson et al., 2019). Sialidase A gene is associated with BV and the presence of biofilm (Hardy et al., 2017b). With the use of BLAST searches, two more *G. vaginalis* sialidases (NanH2 and NanH3) were identified. Regarding the substrate specificity of hydrolysis, recombinant NanH2 and NanH3 were active on Siaα2-3/2-6-linked *N*- and *O*-glycans containing mucosal substrates, whereas recombinant sialidase A showed a limited ability to hydrolyze mucosal and synthetic substrates (Robinson et al., 2019). *G. vaginalis* uses sialidase to hydrolyze sialic acid residue from mucus sialoglycans in the vagina and then catabolizes free carbohydrate, thus contributing to the degradation of vaginal mucus barriers (Lewis et al., 2013). Notably, some *Gardnerella* spp., including *G. swidsinskii*, *G. leopoldii*, and a certain subgroup of *G. vaginalis*, possess negative sialidase activity (Santiago et al., 2011; Vaneechoutte et al., 2019). As for vaginolysin, it is a pore-forming toxic compound belonging to the cholesterol-dependent cytolysin family and facilitates the lysis of target cells, such as vaginal epithelial cells (Gelber et al., 2008; Zilnyte et al., 2015). Other virulence factors, such as prolidase (Cauci et al., 2003) and glycosulfatase (Roberton et al., 2005), are also reportedly associated with BV.

### Biofilms

Biofilm is a structured community of microbes attached to the abiological or biological surface and inlaid in their own secreted polymeric matrix comprising carbohydrate, protein, and nucleic acid (Costerton et al., 1999; Høiby et al., 2011; Flemming et al., 2016). The formation of biofilms is an intricate, dynamic, and interactive process associated with motile planktonic microbes and microbial aggregates (Jung et al., 2017). A variety of bacterial and fungal microbes, such as *Gardnerella* spp. and *Candida* spp., can form biofilms (Machado and Cerca, 2015; Gulati and Nobile, 2016). The biofilms produced by pathogenic microorganisms shape an effective protection against host immune responses and antimicrobials (Swidsinski et al., 2011; Thurlow et al., 2011). The life cycle of biofilms comprises three phases, namely, attachment to a surface, secretion of polymeric matrix and aggregation of microbes for yielding mature biofilms, and dispersion by detaching from biofilms (Machado and Cerca, 2015; Jung et al., 2017).

The polymicrobial biofilms formed on vaginal epithelium play a crucial role in the pathogenesis of BV (Hardy et al., 2017a). *G. vaginalis* is considered the primary colonizer which can establish a scaffold for the attachment of other BV-associated microbes, thus enabling the development of polymicrobial biofilms (Jung et al., 2017). *Atopobium vaginae*, one of the second colonizers of polymicrobial biofilms, is a strict anaerobic microbe with great predictability for BV (Hardy et al., 2016; Castro et al., 2020). *G. vaginalis* biofilms have higher tolerance to two common agents of health vaginal discharge, namely, lactic acid and H_2_O_2_, than planktonic cells (Patterson et al., 2007). This may shelter *G. vaginalis* and other BV-associated microbes from adverse environments. With the use of RNA sequencing, *G. vaginalis* biofilms are observed to have decreased metabolic activity and down-regulated virulence factor (vaginolysin), which are important for biofilm persistence. These phenotypes are probably linked to the recurrent and chronic BV characteristics (Castro et al., 2017). *G. vaginalis* biofilms are reportedly present in fallopian tube and endometrial samples, indicating that *G. vaginalis* biofilms could move to the upper genital tract, thus leading to adverse pregnancy outcomes (Swidsinski et al., 2013).

### Immune Response

BV is considered a dysbiosis often presenting clinical symptoms which can be caused by a large number of microbes with proinflammatory features, coupled with the host immune response (Onderdonk et al., 2016). Vaginal samples from females with BV reportedly harbor high levels of immune mediators, such as interleukin (IL)-8, IL-6, IL-1α, IL-1β, IL-12p70, and TNFα (Platz-Christensen et al., 1993; Hedges et al., 2006; Anderson et al., 2011; Jespers et al., 2017). Different immunological factors might be adopted for various species (Anahtar et al., 2015; Kyongo et al., 2015). For instance, *L. crispatus* is associated with a marked increase in gamma-induced protein 10 (IP-10), and a significant drop in IL-12 (p70), IL-8, IL-1β, and IL-1α. Nevertheless, according to the analysis of vaginal swabs, *G. vaginalis* is correlated with the decline in IP-10 and the increase in IL-12 (p70), IL-8, IL-1β, and IL-1α. Similar to *G. vaginalis*, *A. vaginae* is also associated with the increase and decrease of the same factors (Kyongo et al., 2015). Greater levels of IL-1β, IL-8, and interferon (IFN)-γ have been observed in females harboring a large quantity of *Prevotella* spp. (Anahtar et al., 2015; Kyongo et al., 2015). The critical effect of IL-36G on women with BV is verified. Consequently, IL-36G level increases in vaginal samples of women with BV. IL-36G might play a vital role in the immune response to BV and other diseases (Gardner et al., 2020).

## BV-Associated Diseases

BV is associated with adverse reproductive health outcomes, such as sexually transmitted infections (STIs) and pelvic inflammatory disease (PID) (Unemo et al., 2017; Ravel et al., 2021). Moreover, preterm birth (PTB) (Hillier et al., 1995), low birthweight (Svare et al., 2006), miscarriage (Leitich and Kiss, 2007), and other adverse obstetric outcomes are also linked to BV.

### Sexually Transmitted Infections

STIs are common acute conditions. Although the majority of STIs are generally not fatal, they lead to a large burden of disease. BV increases the risk of some of the STIs, such as herpes simplex virus type 2 (HSV-2), HPV, HIV, and chlamydial, gonococcal, and trichomonal infection. Women with Nugent scores of 9–10 are at the highest risk, whereas women with Nugent scores of 4–8 are at moderate risk of any bacterial STI (*Neisseria gonorrhoeae*, *Chlamydia trachomatis*, and *Trichomonas vaginalis*) (Allsworth and Peipert, 2011). Compared with women with normal vaginal microbiota, women with BV were 4- and 3.4-fold more likely to detect positive for *N*. *gonorrhoeae* and *C*. *trachomatis*, respectively (Wiesenfeld et al., 2003). In a large longitudinal study (n = 3620), BV is reportedly associated with 1.5–2 times elevated risk for the acquisition of chlamydial, gonococcal, or trichomonal infection (Brotman et al., 2010). In another cohort study, temporal associations between STI and BV in both directions were assessed. BV and gonorrhea/chlamydia are risk factors for each other, which indicates treating either condition might have a protective effect on the other (Gallo et al., 2012). Women with BV are at higher risk of acquiring HSV-2 compared with those without BV (Cherpes et al., 2003a; Cherpes et al., 2003b; Cherpes et al., 2005). More recently, several studies have also found that BV is linked to the elevated risk of STIs (Aghaizu et al., 2014; Abbai et al., 2016; Bautista et al., 2017). Unlike the abovementioned observational studies, two randomized trials were performed to investigate whether periodic treatment of BV can affect the incidence of STIs. The first study, which involved women with asymptomatic BV, revealed that the incident chlamydia infection is markedly reduced when intravaginal metronidazole gel was used for 6 months (Schwebke and Desmond, 2007). Another study involving women with BV found that the incidence of *N. gonorrhoeae*, *C. trachomatis*, or *M. genitalium* infection is reduced when intravaginal metronidazole and miconazole were used for 12 months (Balkus et al., 2016).

HIV infection is diagnosed in over 1 million females yearly, and BV is a major risk factor of HIV infection (Klatt et al., 2017). For instance, BV is linked to a greater risk of HIV infection (Taha et al., 1998; Myer et al., 2005), and the level of HIV is greater in the vaginal discharge of women who are infected with HIV and BV than in those of women who are infected with HIV but have no BV (Cu-Uvin et al., 2001; Sha et al., 2005; Elwood et al., 2020). In Uganda, compared with the frequency of HIV-1 in women with normal vaginal flora (14.2%), a markedly elevated frequency (26.7%) was observed in women with severe BV (Nugent scores 9–10) (Ledru et al., 1997). Similarly, a study on HIV-infected Indian women showed the elevated risk of BV in HIV-infected and HPV-positive women and the high prevalence of BV among women infected with HIV (Joshi et al., 2020). Women with HIV^-^BV^+^, HIV^-^BV^-^, HIV^+^BV^-^, and HIV^+^BV^+^ were compared in terms of microbiota of the lower genital tract, and results showed that HIV infection was linked to increased diversity (Spear et al., 2008). Furthermore, vaginal microbial diversity is important in HIV prevention. According to the results of the clinical trial, the efficacy of tenofovir gel microbicide, which prevents HIV transmission, is dependent on vaginal microbiota. Tenofovir decreases the incidence of HIV by 61% in women with *Lactobacillus*-dominant microbiota but merely by 18% in women without *Lactobacillus*. Further, the concentrations of tenofovir are lower in women without *Lactobacillus* and are negatively correlated with BV-associated microbes that could degrade tenofovir by metabolism (Klatt et al., 2017). In addition, compared with women with lower levels of *P. bivia*, women with higher levels (>1%) are linked to 13-fold increased likelihood to become infected with HIV (Cohen, 2016). Women with microbiota dominated by BV-associated microbes have increased amount of HIV target cells, namely CD4 T cells, in the genital mucosa. This may contribute to the elevated HIV acquisition risk (Anahtar et al., 2015; Gosmann et al., 2017).

Among young women, the most common sexually transmitted infection is HPV, which contributes greatly to cervical cancer (Dunne et al., 2007). HPV infection is affected significantly by BV. CST IV-BV is a risk factor for persistent HPV, and the biomarkers of persistent HPV are *Atopobium* spp. and the sialidase gene of *G. vaginalis* (Di Paola et al., 2017). The BV prevalence in the high-risk (HR)-HPV clearing group was 5.0%, which was lower compared with the increased BV prevalence of 11.2% in the HR-HPV persistent group. Further, women who currently have BV reported a lower clearance of HPV than women without BV (Guo et al., 2012). Similarly, BV is reportedly associated with delayed HPV clearance and elevated risk of HPV incident and prevalence (King et al., 2011). In Korea, increased bacterial diversity with decreased *Lactobacillus* spp. was observed in women with HPV. Particularly, BV-associated bacteria, namely *Sneathia* spp., is considered a possible biomarker linked to HPV (Lee et al., 2013). In a study on Nigerian women, a similar linkage was found between the increased proportion of BV-associated bacteria, such as *Leptotrichia* and *Prevotella*, with the decreased in *Lactobacillus* spp. and HR-HPV (Dareng et al., 2016). Among Swedish HPV-infected women, a large proportion of *Megasphaera*, *Prevotella*, *Sneathia*, BVAB1, and BVAB2 was identified in the vaginal microbiota. Women with a total loss of *Lactobacillus* in their vaginal microbiota are 2-fold more likely to have oncogenic HPV (Cheng et al., 2020).

### Pelvic Inflammatory Disease

PID is an infection-caused inflammation of the upper genital tract. BV is considered a risk factor for PID that can cause adverse reproductive sequelae, such as infertility, chronic pelvic pain, and ectopic pregnancy (Brunham et al., 2015). BV-associated microbes were reportedly linked to the elevated risk of PID development, whereas non-BV-associated microbes showed no effect on the risk of PID (Ness et al., 2005). Patients with acute endometritis are more likely to possess BV and less likely to carry lactobacilli (Haggerty et al., 2004). Compared with women with normal vaginal microbiota, subclinical PID is 2.7 times more frequently identified in women with BV (Wiesenfeld et al., 2002). The presence of *A. vaginae*, *S. amnionii*, BVAB1, or *S. sanguinegen*s is associated with PID and its sequelae, including recurrent PID and infertility (Haggerty et al., 2016). In a recent study of women at high risk of STI, the presence of BV-associated microbes, such as *A. vaginae*, *Megasphaera* spp., *Sneathia* spp., *Prevotella amnii*, and *Eggerthella*-like bacterium, in the vagina can increase the likelihood of PID development. Further, a larger bacterial load of BV-associated microbes predicted PID (Haggerty et al., 2020). The identification of BV-associated microbes in PID indicated lower to upper reproductive tract ascension. This finding may be due to the enzymes produced by BV-associated microbes. These enzymes, such as mucinase and sialidase, may degrade mucin barriers and facilitate ascending infection, thereby leading to PID. (McGregor et al., 1994; Soper, 2010).

### Obstetric Outcomes

PTB, which refers to gestation within 37 weeks, has become a serious health challenge worldwide. PTB occurs in about 15 million pregnancies each year and is a primary risk factor of neonatal death (Blencowe et al., 2012; Liu et al., 2016). PTB and other adverse obstetric outcomes have reportedly been associated with BV (Hillier et al., 1995). BV is verified in a meta-analysis to be powerful risk factor for late miscarriage and associated with maternity infectious incidence rate and preterm delivery (Leitich and Kiss, 2007). The BV in early pregnancy is linked to preterm delivery or delivery of an infant with a low birthweight (Svare et al., 2006). High levels of BV-associated microbes, such as *A. vaginae* and *G. vaginalis*, are strongly related to the risk of PTB (hazard ratio 3.3) (Menard et al., 2010). Other BV-associated microbes, such as *Sneathia sanguinegens, Atopobium*, and *Mobiluncus curtsii/mulieris*, are risk factors for spontaneous PTB (Elovitz et al., 2019). A recent multi-omic study of large samples showed that increased level of BV-associated microbes and a sharp decline in the level of *L. crispatus* are observed in women with PTB (Fettweis et al., 2019). As a method of preventing PTB, BV treatment during pregnancy has been evaluated in many studies. However, PTB incidence is not always decreased by antibiotic therapy of BV (Brocklehurst et al., 2013; Shimaoka et al., 2019). BV-associated microbes might cause infection during gestation, because they could move into the uterus before gestation (Goldenberg et al., 2008).

These abovementioned studies highlight the association between BV and many diseases, such as STI, PID, and PTB. BV is a highly prevalent disorder. Thus, the interventions that decrease BV incidence could reduce the incidence of BV-associated diseases. The accurate and efficient diagnosis and treatment of BV may be the key to preventing such diseases.

## Diagnosis and Treatment of BV

### Diagnosis

**Figure 1:**
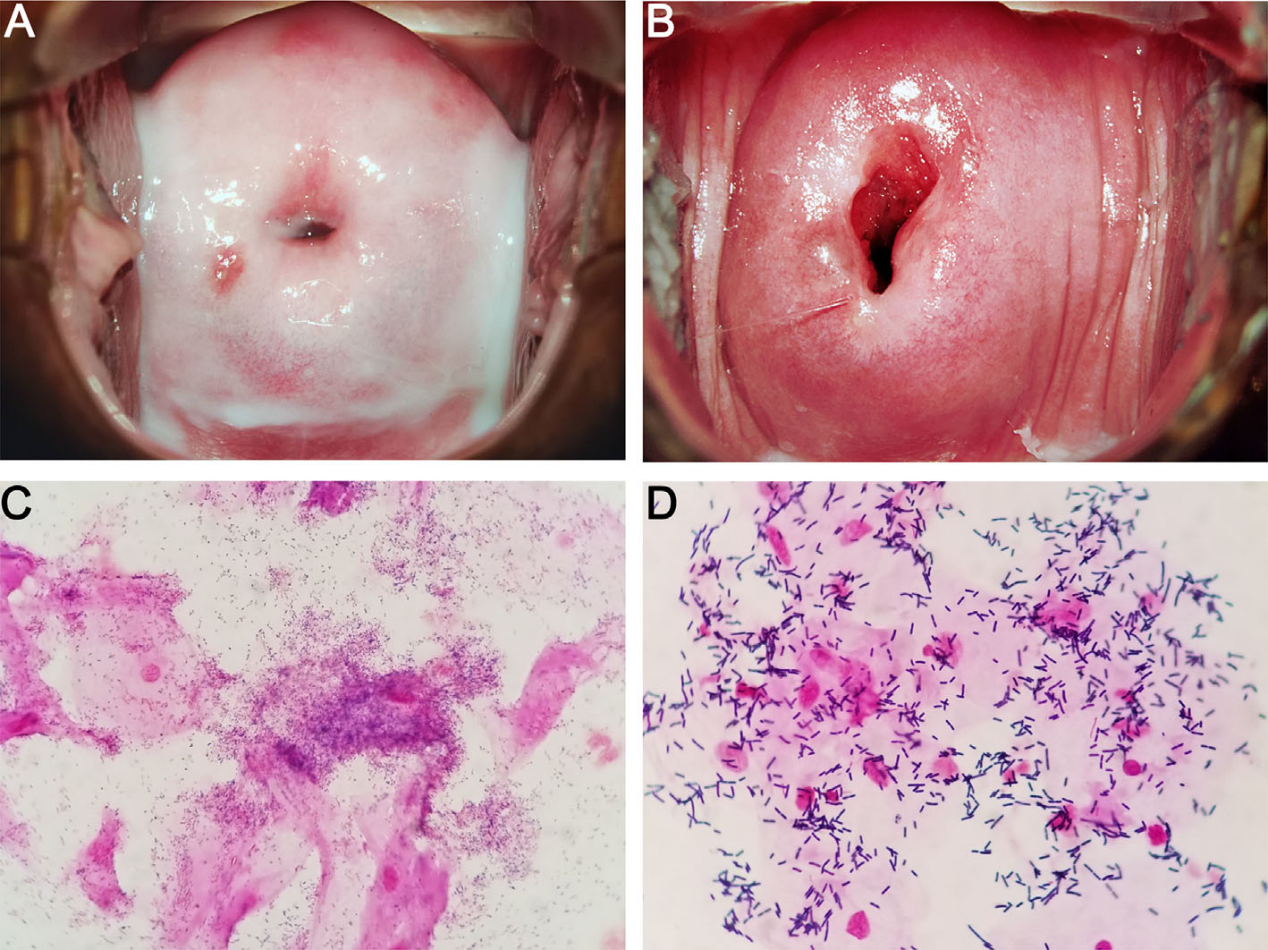
Characteristics of BV **(A, C)** and normal **(B, D)** vaginal microbiome. **(A, C)** show the colposcopic and microscopic examination of BV vaginal discharges, respectively. Homogeneous and milk-like secretions, *Gardnerella*-like microbes, and clue cells are observed. **(B, D)** show the colposcopic and microscopic examinations of normal vaginal discharges, respectively. Homogeneous and clear secretions, *Lactobacillus*-like microbes, and no clue cells are observed.

BV can be diagnosed by performing two standardized tests, namely, Amsel criteria and Nugent score, by using vaginal swabs (Amsel et al., 1983; Nugent et al., 1991). Amsel criteria is used for clinical diagnosis, and at least 3 of the following 4 characteristics are required to determine BV-positive patients: homogeneous and milk–like vaginal fluid (**Figures 1A, B** **)**, increased vaginal pH, fishy odor, and the observation of clue cells (vaginal epithelial cells covered with bacteria) through microscopy (Amsel et al., 1983). Nevertheless, the characteristics above are often absent in some cases, and the corresponding diagnosis is somewhat subjective (Klebanoff et al., 2004). Nugent score relies on the quantitative analysis of the morphotypes of different microorganisms, such as *Lactobacillus* and *Gardnerella*, in Gram-stained vaginal discharge (**Figures 1C, D** **)**. This laboratory-based approach uses a score system, in which the scores of 0–3, 4–6, and 7–10 are considered normal, intermediate, and BV, respectively (Nugent et al., 1991). However, 27% of asymptomatic women possess a high diversity of microbial community dominated by obligate anaerobic bacteria instead of *Lactobacillus* (Ravel et al., 2011). Thus, the combination of the two approaches above with clinical and microbiological morphology findings may result in a more accurate and reliable diagnosis.

There are many point-of-care (POC) methods available for the diagnosis of BV, such as FemExam card, OSOM BVBlue, VGTest ion motility spectrometry (IMS), and wet mount. FemExam card measures amines, proline aminopeptidase, and pH of vaginal discharge (West et al., 2003). The OSOM BV Blue approach is a chromogenic test that detects the level of sialidase in vaginal discharge (Myziuk et al., 2003). VGTest-IMS can be used as an electronic molecular sensor of biogenic amines in vaginal discharge, particularly trimethylamine of BV (Blankenstein et al., 2015). Given the limitations of the aforementioned methods applicable to the diagnosis of BV, attentions have been paid to molecular diagnostic approaches which have the capability to detect and quantify fastidious microbes. Commercially available molecular tests are direct probe tests and nucleic acid amplification tests (Coleman and Gaydos, 2018). Direct probe tests, such as Affirm VP test and bacterial vaginosis/vaginitis panel, apply DNA probe to identify the specific sequences of targeted microbes from vaginal discharge. These tests are most useful for symptomatic women since the targeted microbe for BV, namely *G. vaginalis*, may exist in women with healthy vaginal microbiome (Redelinghuys et al., 2020). Nucleic acid amplification tests include NuSwab, SureSwab BV DNA quantitative real-time PCR, BD Max vaginal panel, and BV panel. NuSwab meartures three positive indicators of BV diagnosis, namely *Megasphaera* type 1, BVAB2, and *A. vaginae*, and one negative indicator of BV diagnosis, namely *L. crispatus* (Cartwright et al., 2012). SureSwab BV real-time PCR is used in the detection of three H_2_O_2_-producing *Lactobacillus* species (*Lactobacillus acidophilus*, *L. jensenii*, and *L. crispatus*) and three BV-associated microbes (*Megasphaera* spp, *A. vaginae*, and *G. vaginalis*) (Coleman and Gaydos, 2018). BD Max vaginal panel can detect two *Lactobacillus* species (*L. jensenii* and *L. crispatus*) and four BV-associated microbes (*A. vaginae*, *G. vaginalis*, BVAB2, and *Megasphaera* type 1) (Gaydos et al., 2017). BV panel identifies *A. vaginae*, *G. vaginalis*, and *Megasphaera* type 1 and 2 as accurate predictors for diagnosis of symptomatic BV (Hilbert et al., 2016).

The etiology of BV remains a persistent conundrum, and consequently, developing and applying more comprehensive, accurate, and advanced approaches for its diagnosis would be necessary. The use of novel approaches, such as deep sequencing of the 16S rRNA gene (Srinivasan et al., 2012), lipidomics (Oliver et al., 2020), glycomics (Wang et al., 2015), metabolomics (Yeoman et al., 2013), and proteomics (Ferreira et al., 2018), provides further insight into the features of BV. Thus, the improved methods will fuel the application of accurate diagnostic strategies and will contribute critically to the development and application of definitive diagnostic methods. For example, based on high-throughput sequencing, women with BV possess heterogeneous vaginal microbiota with high species diversity and richness is observed (Srinivasan et al., 2012). Afterward, with the use of the multi-omic approach (16S rRNA gene sequencing and metabolomics) approach, useful biomarkers (putrescine, cadaverine, 2-methyl-2-hydroxybutanoic acid, and diethylene glycol) are identified for the diagnosis of BV (Yeoman et al., 2013). In addition, artificial intelligence, which refers to machine learning techniques, is performed on the diagnosis of BV and is found to have high interrater reliability and automaticity (Beck and Foster, 2014; Drew et al., 2020).

In short, various diagnostic approaches can be used to diagnose BV. Clinicians should choose a feasible method for BV diagnosis in different cases based on the evaluation of necessary time, cost, and accuracy. For instance, the high-throughput sequencing method might be more suitable for diagnosis among women with recurrent and intractable BV, whereas the wet mount method is widely used for its rapid results and low cost.

### Treatment

Currently, according to Centers for Disease Control and Prevention, the first-line therapeutic strategies are oral metronidazole (500 mg twice a day for 7 days), intravaginal metronidazole gel (5 g once a day for 5 days), and intravaginal clindamycin cream (5 g once a day for 7 days) (Workowski et al., 2015). Metronidazole is a 5-nitroimidazole originally used for the treatment of trichomoniasis (Moffett and Mcgill, 1960) and is found to be useful against anaerobic infection (Tally et al., 1975). Clindamycin is a lincosamide antibiotic that does not differ greatly in terms of effectiveness against BV when compared with metronidazole (Paavonen et al., 2000; Beigi et al., 2004). Vaginal dequalinium is an alternative treatment regimen for BV according to European International Union Against Sexually Transmitted Infections World Health Organization. Dequalinium chloride is a quaternary ammonium compound that possesses cure rates similar to those of clindamycin (Sherrard et al., 2018). Being an agent in the second-line therapeutic strategy, tinidazole has few side effects and good pharmacokinetic profile, and it is also an agent in 5-nitroimidazole class (Dickey et al., 2009; Nyirjesy and Schwebke, 2018). The food and drug administration has recently approved the single-dose of 2 g secnidazole in granule formation for the treatment of BV (Nyirjesy and Schwebke, 2018; Abd El-Aziz et al., 2019). A research has revealed that 2 g secnidazole and 500 mg metronidazole twice a day for 5 days have the same effect (Bohbot et al., 2010). In addition, the single-dose regimen of secnidazole may improve patient compliance. Thus, this next-generation 5-nitroimidazole may serve as a substitute for the 5–7 days therapeutic strategy against BV.

**Figure 2:**
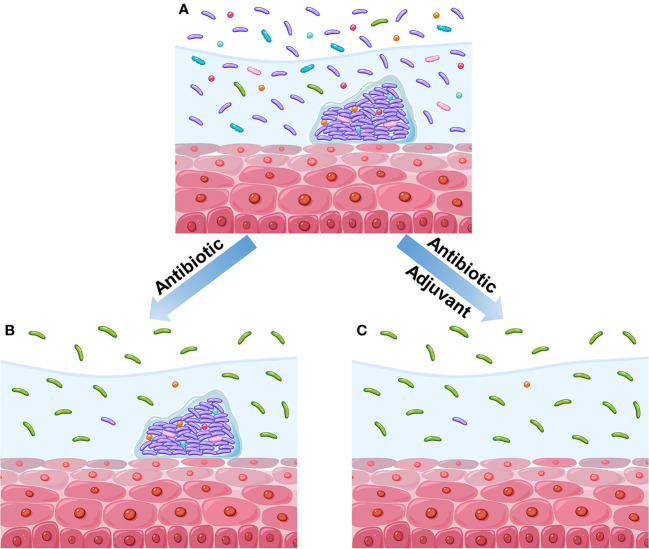
Diagram of the impact of different therapeutic regimens on vaginal microbiome. **(A)** BV vaginal microbiome before treatment. This vaginal microbiome refers to high diversity of microbial community dominated by anaerobic bacteria instead of *Lactobacillus*. Meanwhile, the polymicrobial biofilms are formed on vaginal epithelium. **(B)** Treatment with antibiotics alone reduces the microbial diversity and causes the recovery of *Lactobacillus* population, but the biofilms have not been disrupted. **(C)** Treatment with antibiotics and adjuvant reduces the microbial diversity and causes the recovery of *Lactobacillus* population; moreover, the biofilms have been disrupted.

Although these antibiotics are effective against BV-associated bacteria and somewhat relieve symptoms, the remission is usually temporary, and many patients relapse after treatment (Bradshaw et al., 2006; Hay, 2009; Mayer et al., 2015). The high recurrence rate (50%–67%) may be the result of the inability of antibiotics to eliminate the biofilm-associated bacteria of BV in vagina (**Figure 2**) (Swidsinski et al., 2008; Swidsinski et al., 2011; Javed et al., 2019; Ahrens et al., 2020; Verwijs et al., 2020). For example, Ahrens et al. reported that *G. vaginalis* and other BV-associated bacteria are eradicated or are largely decreased in 58% of the patients treated with metronidazole. Meanwhile, none of these bacteria are eliminated in the remaining half of the patients. This phenomenon is attributed to the sheltering of *G. vaginalis* and other bacteria by biofilms (Ahrens et al., 2020). In addition, Swidsinski et al. observed the persistence of *G. vaginalis* biofilms after oral metronidazole therapy (Swidsinski et al., 2008). Therefore, a promising therapeutic strategy against BV, i.e., adjuvant-based therapy with the ability to disrupt biofilms, has been developed (**Figure 2**). Extracellular DNA is an important integrant of *G. vaginalis* biofilms. The enzymatic activity of DNase can be used to disrupt newly formed and mature biofilms. DNase has shown its ability to release microorganisms from biofilms to supernatant fractions and to enhance the activity of metronidazole (Hymes et al., 2013). Similarly, lysozyme can reportedly disrupt biofilms and prevent their formation. The combination of lysozyme and antibiotic (clindamycin or metronidazole) can potentiate both the ability of antibiotics and biofilm disruption (Thellin et al., 2016). In addition, the use of amphoteric tenside sodium cocoamphoacetate causes degradation of the established biofilms and enhances the effect of metronidazole (Gottschick et al., 2016). Lauramide arginine ethyl ester and subtilosin reportedly have great bactericidal effect on *G. vaginalis* biofilms (Turovskiy et al., 2012). These two compounds have displayed a synergistic effect with antibiotics (clindamycin and metronidazole) against *G. vaginalis* biofilms (Algburi et al., 2015). Besides the abovementioned adjuvants investigated with antibiotics, many others have been studied individually. For instance, *L. reuteri* RC-14 can degrade surface density, depth, and area of *G. vaginalis* biofilms (Saunders et al., 2007). Thymol is effective in the inhibition of mature and native *G. vaginalis* biofilms (Braga et al., 2010). Endolysin (PM-477) can selectively and effectively eliminate *Gardnerella* in native polymicrobial biofilm and in cultures of isolated strains (Landlinger et al., 2021). Other strategies involving the utilization of boric acid (Reichman et al., 2009), octenidine (Swidsinski et al., 2015), cationic amphiphiles (Algburi et al., 2017; Weeks et al., 2019), and amphoteric tenside (WO3191) also reportedly have a positive effect on BV-associated biofilms (Gottschick et al., 2017).

Microbial-based therapeutics have recently attracted an increasing amount of interest owing to the beneficial effects to the host health. As pivotal bacteria in the healthy vaginal microbiome, *Lactobacillus* species can act as antimicrobial adjuvants due to their ability to potentiate the effect of antibiotics (Larsson et al., 2011; Bodean et al., 2013; Recine et al., 2016; Bohbot et al., 2018; Cohen et al., 2020). In 2020, a phase 2b trial (NCT02766023) was conducted on 228 women to assess the efficiency of *L. crispatus* CTV-05 in preventing BV relapse. When *L. crispatus* CTV-05 was used after antibiotic treatment, a remarkably lower rate of BV recurrence was observed at 3 months compared with placebo (Cohen et al., 2020). Vaginal microbiota transplantation (VMT), in which optimum vaginal microbiota is transplanted to patients, is another microbial-based therapeutic strategy. In 2019, the first exploratory research has reported the viability of utilizing VMT as a long-term therapeutic regimen for women with intractable BV (Lev-Sagie et al., 2019). In this study, five patients with intractable BV were treated with VMT after antibiotic treatment, and long-lasting relief was shown in four of these patients in the follow-up period of 5–21 months after VMT. This finding suggested the remarkable alleviation of symptoms and the rebuilding of the vaginal microbiota with *Lactobacillus* spp. dominance. The risks of this treatment include the acquisition of antimicrobial-resistant microbes, sperms, undetected pathogens, and other clinically silent phenotypes from the donors. Overall, antibiotics, biofilm-disrupting agents, probiotic *Lactobacillus*, and VMT can be utilized separately or in combination to regulate the microbiome through the reestablishment of vaginal eubiosis.

The intricate and dynamic vaginal microbiome brings the challenge for diagnosis. The challenge for treatment is attributed to inaccurate diagnostics, biofilm, antibiotic resistance, and the simultaneous elimination of both pathogenic bacteria and probiotics, such as *Lactobacillus.* It is expected that future research should be conducted to target specific microbes, thus eliminating more pathogenic bacteria, but without affecting probiotic microorganisms. In addition, it is necessary to develop personalized diagnosis and treatment by taking individual differences in vaginal microbiome into consideration.

Further, with increasing knowledge of BV-associated epidemiology and pathogenesis, more improved novel therapeutic strategies will be developed in the future.

## Conclusion

The vaginal microbiome forms a homeostatic and mutualistic relationship with human host and plays an important role in vaginal health and disease. The variations of internal and/or external factors lead to the breakdown of a balanced ecosystem, which is also known as dysbiosis. Although the increasing scientific knowledge has already provided insights into the characteristics of vaginal microbiome and its correlation with diseases, such as STIs, PID, PTB, there is still inadequate understanding of interactions between microbiota and the host. Efforts should be made to reveal the mechanism of interactions between species and their impact on vaginal microbiome.

As a highly prevalent dysbiosis, BV triggers numerous adverse health outcomes and becomes a burden to individuals and public health. *G. vaginalis* is the most common microorganism detected in BV, but its presence in the vagina does not always lead to BV. Other microorganisms, such as *Atopobium* and *Prevotella*, also have a strong relationship with BV. These BV-associated microbes can affect immune mediators, which may serve as predictive biomarkers for dysbiosis, in the vaginal environment. Although the epidemiology and pathogenesis of BV is not fully understood, studies based on genomic, lipidomic, glycomic, metabolomic, and proteomic techniques may provide further insights. More improved novel and accurate diagnosis and therapeutic strategies will be developed based on the accumulated information on BV.

## Author Contributions

XC drafted the manuscript for publication. YL participated in writing chapter diagnosis and treatment of BV. TC prepared the first draft of **Figure 1**. RL reviewed and revised the manuscript. All authors contributed to the article and approved the submitted version.

## Funding 

This work was supported by Science and Technology Project of Jinan Municipal Health Commission (2019-1-25 and 2020-4-71).

## Conflict of Interest

The authors declare that the research was conducted in the absence of any commercial or financial relationships that could be construed as a potential conflict of interest.
